# IgE Production to α-Gal Is Accompanied by Elevated Levels of Specific IgG1 Antibodies and Low Amounts of IgE to Blood Group B

**DOI:** 10.1371/journal.pone.0055566

**Published:** 2013-02-04

**Authors:** Theo Rispens, Ninotska I. L. Derksen, Scott P. Commins, Thomas A. Platts-Mills, Rob C. Aalberse

**Affiliations:** 1 Sanquin Research and Landsteiner Laboratory, Academic Medical Centre, University of Amsterdam, The Netherlands; 2 Division of Allergy and Immunology, University of Virginia, Charlottesville, Virginia, United States of America; Agency for Science, Technology and Research - Singapore Immunology Network, Singapore

## Abstract

IgE antibodies to gal-α-1,3-gal-β-1,4-GlcNAc (α-gal) can mediate a novel form of delayed anaphylaxis to red meat. Although IgG antibodies to α-gal (anti-α-gal or anti-Gal) are widely expressed in humans, IgE anti-α-gal is not. We explored the relationship between the IgG and IgE responses to both α-gal and the related blood group B antigen. Contradicting previous reports, antibodies to α-gal were found to be significantly less abundant in individuals with blood group B or AB. Importantly, we established a connection between IgE and IgG responses to α-gal: elevated titers of IgG anti-α-gal were found in IgE-positive subjects. In particular, proportionally more IgG1 anti-α-gal was found in IgE-positive subjects against a background of IgG2 production specific for α-gal. Thus, two types of immune response to α-gal epitopes can be distinguished: a ‘typical’ IgG2 response, presumably in response to gut bacteria, and an ‘atypical’, Th2-like response leading to IgG1 and IgE in addition to IgG2. These results suggest that IgE to a carbohydrate antigen can be formed (probably as part of a glycoprotein or glycolipid) even against a background of bacterial immune stimulation with essentially the same antigen.

## Introduction

IgE represents the class of antibodies that mediate hypersensitivity to a variety of allergens. Typically, allergens are proteinaceous in nature and may induce formation of IgG antibodies as well as IgE antibodies. Nevertheless, IgE may also be directed to carbohydrate structures. In particular, so-called cross-reactive carbohydrate determinants (CCD) can be targets for IgE responses [1], but the role of anti-CCD in triggering allergic symptoms is unclear [2]. However, it was recently found that some individuals can elicit an IgE response to the α-gal epitope [3]; [4]. Patients with pre-formed IgE antibodies to α-gal were found to suffer from anaphylactic reactions upon first exposure with cetuximab, a chimeric therapeutic antibody with murine variable domains that contains the α-gal epitope [5]. Moreover, cases of meat allergy have also been linked to IgE anti-α-gal [6]–[8].

**Figure 1:**
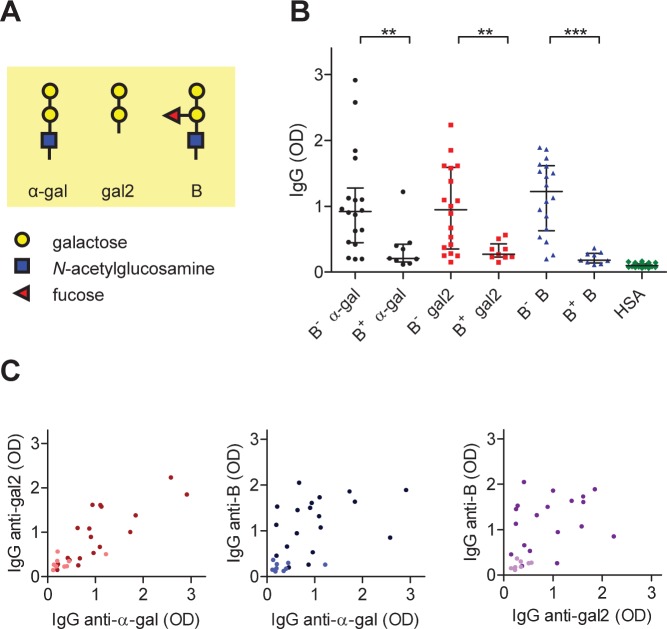
IgG responses to α-gal and blood group B antigens. A) Schematic representations of gal-α-1,3-gal-β-1,4-GlcNAc (α-gal), gal-α-1,3-gal (gal2) and blood group B (B) antigens. B) IgG anti-α-gal, anti-gal2, and anti-B in B^+^ and B^–^ individuals, as measured in ELISA (1 µl serum/test). Bars represent median and interquartile ranges. B^–^ individuals make significantly more IgG to all three antigens (Mann-Whitney; ** *p*<0.01; *** *p*<0.001). C) Correlations between IgG anti-α-gal and IgG anti-gal2 (Spearman *r* = 0.83; *p*<0.0001), IgG anti-α-gal and IgG anti-B (*r* = 0.59; *p* = 0.009), and IgG anti-gal2 and IgG anti-B (*r* = 0.58; *p* = 0.0011). Light-colored dots correspond to B^+^ subjects. None of the sera contained detectable amounts of IgE anti-α-gal.

The IgG counterpart of IgE anti-α-gal are natural antibodies found in all humans. These antibodies are presumably produced in response to gut microorganisms [9]; [10], and were found to contribute to xenograft rejection [11]. It was reported that these antibodies are widely expressed by humans regardless of ABO blood type and constitute up to ∼ 1% of all circulating IgG [12]; [13], although this value was disputed by others [14]; [15]. The α-gal epitope is structurally closely related to the blood group B antigen (Figure 1A) and first recognized by Landsteiner as ‘B-like’ antigen on rabbit erythrocytes [16]. Therefore, antibody responses to both antigens are expected to be related. Indeed, whereas α-gal specific antibodies were found in B-positive individuals, antibodies that cross-react with B antigen were observed in B-negative individuals [13]. However, a quantitative relation between anti-B and anti-α-gal antibodies has not been established.

It was recently shown that IgE anti-α-gal can be induced by tick bites in certain regions of the USA including Virginia [17]. Furthermore, in parasite-infected patients from central Africa, IgE anti-α-gal was also demonstrable [4]. In this respect, it is of interest that IgG anti-α-gal was found to be elevated in cases of leishmaniasis [18]. However, no data exist on the relationship between IgE and IgG responses to α-gal. Often, IgG antibodies to carbohydrates are largely IgG2 [19]–[21] – as was reported for anti-α-gal [14]. Anti-carbohydrate IgG2 antibodies are thought to be produced in a T-cell independent manner. On the other hand, switch to IgE is dependent on Th2 associated cytokines (IL-4, IL-13) [22]. It is possible that IgE anti-α-gal is produced via a T cell independent pathway. Alternatively, the IgE response is an independent response to the same antigen, but presented to the immune system in a different context, i.e., accompanied by signals that trigger a Th2 response leading to IgE. Of course, a Th2 response characterized by IgE may also have developed *instead of* the usual formation of ‘natural’ anti-α-gal antibodies.

Here we explored the relationship between the IgG and IgE responses to both α-gal and blood group B. We measured IgG and IgG subclasses to α-gal and B in subjects that did or did not produce IgE antibodies to α-gal. We also included the terminal gal-α-1,3-gal disaccharide common to both carbohydrate antigens in the analysis. Our results indicate that the IgE anti-α-gal response is characterized by elevated IgG anti-α-gal, particularly IgG1, against a background of IgG2 production.

## Materials and Methods

### Sera/Ethics Statement

Sera were obtained under written informed consent from subjects (n = 20) from Virginia that were previously tested positive for IgE to α-gal [17], and healthy volunteers without detectable IgE to α-gal from both Virginia, USA (n = 9) and Amsterdam, The Netherlands (n = 27). Approval was obtained from University of Virginia Human Investigation Committee (IRB 13298).

### Materials

Gal-α-1,3-Gal-β-1,4-GlcNAc-human serum albumin (NGP2334, hereafter referred to as α-gal-HSA), Gal-α-1,3-Gal-HSA (NGP2203, hereafter referred to as gal2-HSA, blood group B-HSA (NGP9323), and Gal-α-1,3-Gal-β-1,4-GlcNAc trisaccharide (GN334) were obtained from Dextra laboratories, Reading, UK. Horseradish peroxidase (HRP)-labeled monoclonal antihuman IgG (MH-16), IgG1, (clone HP6188, MH161-1) IgG3 (clone HP6095, MH163-1) or IgG4 (clone HP6196, MH164-4) were obtained from Sanquin, The Netherlands; HRP-labeled mouse monoclonal antibody to human IgG2 (clone HP6002) was obtained from Abcam, Cambridge, UK.

### IgE Radioallergosorbent Test

Serum IgE to α-gal and blood group B were measured by a radioallergosorbent test (RAST) using α-gal-HSA or blood group B-HSA coupled to Sepharose and detected by radiolabeled anti-IgE essentially as described before [23]. 1 mg of α-gal-HSA or blood group B-HSA was coupled to 100 mg of CNBr-activated Sepharose (Amersham Biosciences, Uppsala, Sweden). Ten microliters of serum was incubated with 0.5 mg Sepharose in a total volume of 1 ml of PBS-AT (PBS, pH 7.4, supplemented with 0.3% bovine serum albumin, 0.1% Tween-20, 10 mM EDTA and 0.05% (w/v) NaN_3_) and incubated overnight on a vertical rotor. After washing five times with PBS-T (PBS supplemented with 0.1% Tween-20 and 0.05% (w/v) NaN_3_), ^125^I-labeled anti-IgE was added and after overnight incubation and washing, radioactivity was measured. The results were expressed as arbitrary units as compared with known standards.

### IgG ELISA

To measure IgG antibodies to the carbohydrate antigens maxisorp ELISA plates were coated overnight at room temperature with α-gal-HSA (1 µg/ml), gal2-HSA (2 µg/ml), blood group B-HSA (1 µg/ml), or HSA (2 µg/ml; Albuman, Sanquin, The Netherlands) in PBS. After five times washing with PBS supplemented with 0.02% Tween-20 (PBS-T), plates were incubated for 1 h at room temperature with serum sample (0.03–1 µl/test) diluted in PBS supplemented with 0.02% Tween-20 and 0.1% HSA (PBS-HT) (100 µl final volume). Plates were washed five times with PBS-T. Then 100 µl of 1 µg/ml antihuman IgG-HRP (MH-16) in PBS-HT was added followed by incubation for 1 h at room temperature. Plates were washed five times with PBS-T, and 100 µl of TMB substrate (100 µg/ml) and 0.003% (v/v) hydrogen peroxide in 0.11 M sodium acetate buffer (pH 5.5) was added to each well. After 10 minutes the reaction was stopped by the addition of 2 M H_2_SO_4_. Absorbance was measured at 450 nm. OD values were normalized to readings of a reference serum that was included on each plate.

### IgG Subclass ELISA

To measure IgG subclasses to the carbohydrate antigens maxisorp ELISA plates were coated and incubated with serum as described above. Then 100 µl of antihuman IgG1-HRP (0.1 µg/test), antihuman IgG2-HRP (0.4 µg/test), antihuman IgG3-HRP (0.2 µg/test), or antihuman IgG4-HRP (0.1 µg/test) in PBS-HT was added followed by incubation for 1 h at room temperature, and further developed as described above. In order to obtain approximate values for the relative proportions of the subclasses, OD for the respective subclasses were compared to a standard consisting of monoclonal IgG1, 2, 3, or 4 antibodies titrated onto a coat of an anti-human kappa antibody (K35, Sanquin, The Netherlands) and detected with the respective anti-subclass antibody. Relative concentrations of the monoclonal antibodies were based on the same titrations but with anti-human IgG (MH-16) detection.

### Statistical Analysis

All data analysis was carried out using Graphpad Prism 5. Single comparisons were made using Mann-Whitney’s test; multiple comparisons with Kruskal-Wallis with Dunn’s multiple comparison test; (non-parametric) correlations are expressed as Spearman *r*. Details are indicated in Figure legends.

## Results

### IgG Antibodies to α-gal and B in B^+^ and B^–^ Individuals

To elucidate the relationship between IgG antibody responses to α-gal and blood group B antigens, we first tested sera from healthy volunteers for antibodies to either the α-gal trisaccharide, the terminal disaccharide gal-α-1,3-gal (hereafter termed gal2), or blood group B tetrasaccharide, as depicted in Figure 1A. We observed substantial variation in IgG to all three antigens in B^–^ individuals (Figure 1B). By contrast, B^+^ individuals not only have low titers of IgG anti-B, as expected, but also of anti-α-gal and anti-gal2, in all cases significantly less than the B^–^ group: median OD_1 µl_ (IQR) were B^–^: 0.92 (0.45–1.28) vs B^+^: 0.21 (0.15–0.42) for α-gal (*p* = 0.007), B^–^: 0.95 (0.35–1.59) vs B^+^: 0.27 (0.23–0.43) for gal2 (*p = *0.006), and B^–^: 1.22 (0.63–1.61) vs B^+^: 0.18 (0.14–0.29) for B (*p = *0.0002). Apparently, expression of the B epitope predisposes to making low titers of anti-α-gal. Antibody responses to α-gal correlate well with anti-gal2 (Figure 1C, Spearman *r* = 0.83). Correlation between either anti-α-gal or anti-gal2 with anti-B is much less (resp. *r* = 0.59 and 0.58). Thus, it appears that the presence of blood group antigen B induces tolerance to the B antigen and generally eliminates most B cell clones that could otherwise have reacted with α-gal, although in individual cases, a B^+^ subject can still make considerable amounts of IgG anti-α-gal. In B^–^ subjects no tolerance to B develops, but the antibody responses to α-gal and B are nevertheless only poorly quantitatively linked, suggesting partially independent antibody responses to these antigens.

### IgE Antibodies to α-gal Correlate with Raised IgG Antibodies to α-gal but not B

**Figure 2:**
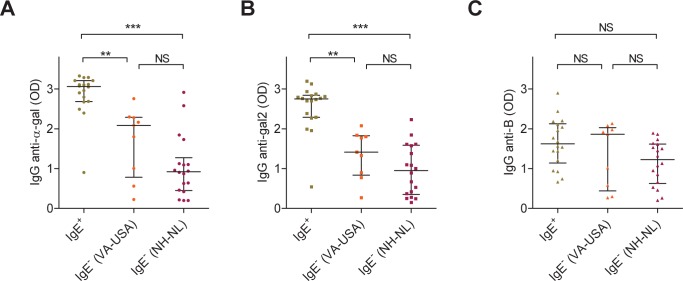
Individuals positive for IgE anti-α-gal make significantly more IgG anti-α-gal. IgG antibody responses to A) α-gal, B) gal2, and C) B antigens in individuals who are positive for IgE anti-α-gal (IgE^+^), and individuals from both Virginia, USA, and North-Holland, The Netherlands, who are negative for IgE anti-α-gal (IgE^–^), as measured in ELISA (1 µl serum/test). All subjects are B^–^. IgE^+^ individuals make significantly more IgG anti-α-gal and anti-gal2, but not anti-B (Kruskal-Wallis with Dunn’s multiple comparison test). NS: not significant.

Next, we compared IgG responses between B^–^ subjects that did or did not express IgE antibodies to α-gal (none of the B+ subjects were found to express IgE anti-α-gal). This selection was made based on a radioimmunoassay for IgE to α-gal-HSA (Figure S1). Anti-α-gal IgE^+^ and IgE^–^ subjects from Virginia, USA, as well as IgE^–^ subjects from North-Holland, The Netherlands were included. We found that IgG anti-α-gal and anti-gal2, but not anti-B were significantly elevated in the anti-α-gal IgE^+^ group as compared to both IgE^–^ groups (Figure 2). No significant differences were observed between anti-α-gal IgE^–^ subjects from Virginia or North-Holland. In other words, in IgE^+^ subjects, IgG to α-gal, but not B, is elevated, indicating a distinct immune response that leads to both enhanced IgG production as well as IgE production.

**Figure 3:**
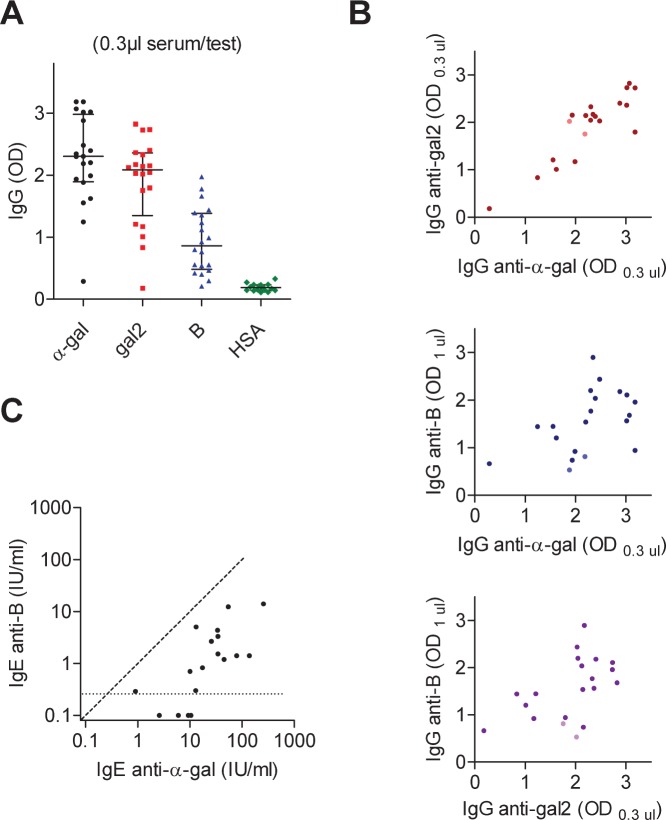
Correlations of IgG and IgE antibody responses in subjects positive for IgE anti-α-gal. A) IgG anti-α-gal, anti-gal2, and anti-B in IgE^+^ subjects (0.3 µl serum/test), including two B^+^ cases. B) Correlations between IgG anti-α-gal and IgG anti-gal2 (Spearman *r* = 0.76; *p*<0.0001), IgG anti-α-gal and IgG anti-B (*r* = 0.48; *p* = 0.048), and IgG anti-gal2 and IgG anti-B (*r* = 0.53; *p* = 0.021). Light-colored dots correspond to B^+^ subjects. C) IgE anti-α-gal and anti-B measured in IgE radioimmunoassay. Median responses were 21.7 (IQR 9.9–47.5) IU/ml for IgE anti-α-gal, and 1.3 (IQR 0.3–3.6) IU/ml for IgE anti-B, which is significantly lower (*p*<0.0001). Levels are nevertheless correlated (*r* = 0.76; *p* = 0.0003).

Antibodies to α-gal and gal2 were also tested at a higher serum dilution for the IgE^+^ group (Figure 3A), and as in case of IgE^–^ subjects, a good correlation (Spearman *r* = 0.76; *p*<0.0001) was found between anti-α-gal and anti-gal2, and weak correlations between either anti-α-gal (*r* = 0.48; *p* = 0.048) or anti-gal2 (*r* = 0.53; *p* = 0.021) with anti-B (Figure 3B). Furthermore, IgE anti-B was measured in the IgE^+^ group (Figure 3C). IgE anti-B was detected in some cases, albeit significantly lower in titer: 21.7 (IQR 9.9–47.5) IU/ml for IgE anti-α-gal, and 1.3 (IQR 0.3–3.6) IU/ml for IgE anti-B (*p*<0.0001). Titers are nevertheless correlated (*r* = 0.76).

### IgG Subclasses to α-gal and B

**Figure 4:**
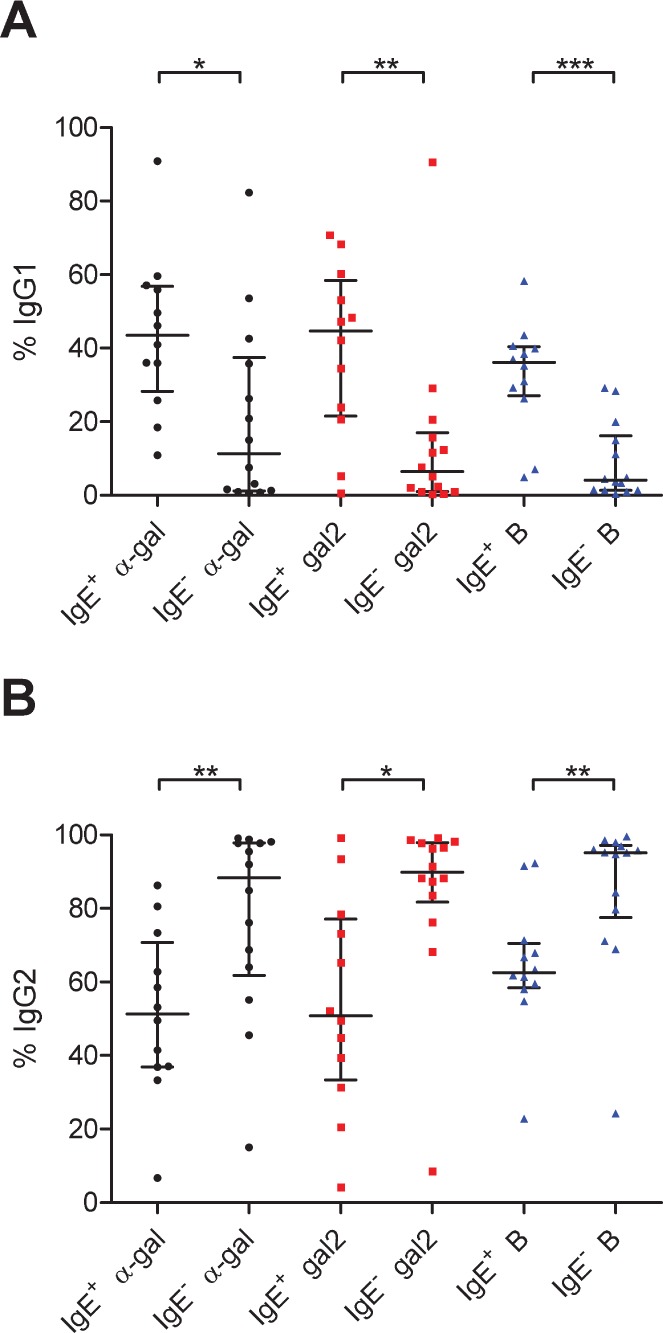
IgG subclasses to α-gal, gal2, and B antigens in IgE^+^ and IgE^–^ individuals. Contribution of A) IgG1 and B) IgG2 antibodies to α-gal, gal2, and B antigens in IgE^+^ and IgE^–^ individuals from both Virginia and North-Holland. Bars represent median and interquartile ranges. IgE^+^ individuals make significantly more IgG1 to all three antigens (Mann-Whitney; * *p*<0.05; ** *p*<0.01; *** *p*<0.001).

To obtain more insight in the seemingly distinct IgG responses to α-gal that are characterized by the presence or absence of specific IgE antibodies, IgG subclasses were measured in both IgE^+^ and IgE^–^ subjects (selected for OD to all three antigens greater than 0.8). Approximate proportions of the subclass distributions were calculated by comparison to reference curves as described in Materials and Methods. In most IgE^–^ subjects, the majority of anti-α-gal antibodies are IgG2, as expected (Figure 4). In many IgE^+^ subjects on the other hand, a substantial fraction of anti-α-gal is IgG1, in line with a Th2-like antibody response [22]. A similar preferential production of IgG1 in the IgE^+^ group is observed for anti-gal2 and anti-B. In absolute terms, IgG2 to α-gal and gal2, but not B, is elevated in the IgE^+^ group, but the fold increase in IgG1 is much larger compared to IgG2 (Figure S2). IgG3 antibodies were only occasionally found, and IgG4 was detectable only in small amounts (<1% of IgG antibody) in IgE^+^ subjects (Figure S3).

## Discussion

In the first studies of Galili addressing the prevalence and specificities of anti-α-gal antibodies invariably rabbit erythrocyte agglutination were used as read-out [12]; [13]; [24]. In those studies, anti-α-gal antibodies were found to be present in remarkably constant titers (ca. 1% of total IgG) in >95% of a healthy population regardless of ABO blood type. To our knowledge, these findings have so far not been confirmed in assays using well-defined carbohydrate antigens. On the contrary, a much wider variation in titers was observed using ELISA tests that made use of synthetic carbohydrates [14]; [15]; [25]; [26]. We used synthetic sugar antigens coupled to HSA to assess antibody levels to α-gal. Specificity of the α-gal ELISA was demonstrated by the low background signals on an HSA coat (Figure 1B), and by the ability to inhibit the IgG binding by soluble α-gal trisaccharide (Figure S4). In contrast to the reports by Galili, but in agreement with other studies [26]; [27], our results imply that B cell responses to α-gal and B are correlated: tolerance to B results in low levels of anti-α-gal. Nevertheless, in B^–^ individuals, antibody responses to α-gal and B appear to be poorly quantitatively correlated, and may derive in part from different B cell populations that were triggered independently. This becomes even more pronounced in those subjects in which both IgG and IgE anti-α-gal is elicited largely independently of antibodies to B: IgG as well as IgE to α-gal, but not to B, are significantly raised. At the same time, some IgE that binds to bloodgroup B antigen is also induced, and the contribution of IgG1 antibodies to B is significantly larger in individuals who are also producing IgE anti-α-gal.

Our results indicate that the IgE anti-α-gal response as induced by tick bites is characterized by elevated IgG anti-α-gal, particularly IgG1, against a background of IgG2 production. Antibody responses to allergens often involve IgG1 antibodies (and sometimes IgG4) in addition to IgE antibodies [28]. The IgE anti-α-gal response therefore appears to be part of a Th2 type immune response. As such, we expect that if a subject needs to be desensitized, in principle, established protocols of immunotherapy may be of use, although cross-reactivity to blood group B antigen should be taken into account. It is also relevant to point out that although class switch from IgG2 to IgE is theoretically possible, switch from IgG2 to IgG1 is not, as a consequence of the sequence order in which the genes for the isotypes are arranged on the chromosome. Probably, both IgG1 and IgE are predominantly formed by class switch from non-switched (IgM) B cells.

Raised IgG anti-α-gal was previously found in a number of conditions, including Chagas’ disease, american leishmaniasis, and malaria[18]; [24]; [29]. In parasitic infections, IgE production may be anticipated, and IgE anti-α-gal has indeed been found in parasite-infected subjects [4]. Therefore, the direct link between IgG upregulation and IgE production as demonstrated in the present study is important and suggests that previously reported conditions of elevated IgG anti-α-gal[18]; [24]; [29] can be expected to be a risk factor for production of IgE anti-α-gal. Most anti-carbohydrate antibodies have relatively low avidity. Signals in both the ELISA and the RAST will therefore typically reflect concentration as well as avidity – not unlike the capacity of these antibodies to bind to the same epitopes on e.g. cetuximab. Thus, the specific increase in reactivity may reflect a combination of affinity maturation (during a T-cell dependent antibody response) and increased antibody concentration.

Correlation between binding to α-gal and gal2 was good. It appears that binding to α-gal is largely determined by the terminal galactoses, as has been suggested by others [28]; [30]. Specificity to α-gal on the other hand may be also determined by the proximal sugar residues of α-gal and related sugar epitopes, B in particular. The specificity is probably largely due to exclusion of binding to the related epitopes (repulsive interactions) rather than enhanced binding to the primary epitope. Of note, binding to α-gal on therapeutic antibodies is also dependent on the structural environment of the epitope: Fc-bound α-gal on infliximab was not recognized by IgE anti-α-gal, probably due to sterical interference by the polypeptide structure surrounding the glycan. This was not an intrinsic property of the carbohydrate bearing α-gal, because after proteolytic digestion of infliximab binding to residual carbohydrate moiety could be demonstrated [23].

IgG antibodies to blood group antigens A and B are known to be largely IgG2 in most cases. This explains why they usually cause little problems in the unborn fetus: although able to cross the placenta, IgG2 antibodies are poor triggers of the effector functions of the immune system. In this respect, the finding that IgG antibodies to B can be of the IgG1 subclass in certain populations may be of relevance. It remains to be investigated if these antibodies may have any implications during pregnancy.

It appears that ‘normal’ exposure to α-gal does not lead to IgE production. At the same time, this established immune response to α-gal appears to essentially offer no protection against induction of IgE following ‘atypical’ exposure to α-gal (presumably helminth or ectoparasites exposure). IgG2 antibodies are weak activators of effector functions and theoretically may serve as ‘blocking antibodies’, similar to IgG4 [31]; [32]. However, cases of anaphylaxis triggered by α-gal [3]; [4] suggest that prevention of allergic symptoms via these ‘blocking antibodies’ is ineffective. While this may not be a general characteristic of specific IgG antibodies, it does fit with a recent study in which it was found that IgG4 antibodies against rodents in laboratory animal workers do not protect against allergic sensitization [33].

In summary, the IgE anti-α-gal response is characterized by elevated IgG anti-α-gal, particularly IgG1, and low amounts of IgE anti-B, against a background of IgG2 production. The results indicate that IgE to a carbohydrate epitope can be formed even against a background of bacterial immune stimulation with essentially the same antigen.

## Supporting Information

Figure S1 **IgE antibodies to α-gal.** IgE antibodies to α-gal as measured in a radioimmunoassay of sera from Virginia, USA, originally tested positive (IgE^+^) or negative (IgE^–^ VA, USA) for cetuximab as well as sera from healthy volunteers from North-Holland, The Netherlands, who were all found to be negative for IgE antibodies to α-gal (IgE^–^ NH, NL).
(EPS)

Figure S2 **IgG1 and IgG2 antibodies to α-gal, gal2, and B antigens in IgE^+^ and IgE^–^ individuals.** Individuals from both Virginia and North-Holland were included. A) IgG1 and B) IgG2. Bars represent median and interquartile ranges. IgE^+^ individuals make significantly more IgG1 to all three antigens, and significantly more IgG2 to α-gal and gal2 (Mann-Whitney; * *p*<0.05; ** *p*<0.01; *** *p*<0.001).
(EPS)

Figure S3 **IgG subclass distribution to α-gal, gal2, and B in individual sera.** Examples of calibration curves shown in upper panels.
(EPS)

Figure S4 **Inhibition of IgG antibodies binding to α-gal-HSA coat with Gal-α-1,3-Gal-β-1,4-GlcNAc trisaccharide.**
(EPS)
